# Novel Senecavirus A in Swine with Vesicular Disease, United States, July 2015

**DOI:** 10.3201/eid2207.151758

**Published:** 2016-07

**Authors:** Baoqing Guo, Pablo E. Piñeyro, Christopher J. Rademacher, Ying Zheng, Ganwu Li, Jian Yuan, Hai Hoang, Phillip C. Gauger, Darin M. Madson, Kent J. Schwartz, Paisley E. Canning, Bailey L. Arruda, Vickie L. Cooper, David H. Baum, Daniel C. Linhares, Rodger G. Main, Kyoung-Jin Yoon

**Affiliations:** Iowa State University, Ames, Iowa, USA

**Keywords:** Senecavirus A, Seneca Valley virus, vesicular disease, swine, exhibition, market hog, USA, United States, viruses

**To the Editor:** Senecavirus A (SVA; formerly known as Seneca Valley virus [SVV] belongs to the genus *Senecavirus*, family *Picornaviridae* (*1*,*2*). SVA was first isolated in 2001 as a contaminant of the PER.C6 cell line and designated as SVV-001 (*1*,*3*). Since its discovery, SVA has been infrequently detected in swine with idiopathic vesicular disease (IVD) (*4**–**6*), which clinically resembles foot-and-mouth disease, swine vesicular disease, vesicular exanthema of swine, and vesicular stomatitis. The virus has also been retrospectively detected in previous cases with various clinical conditions in the United States during 1988–2001 (*7*). However, the clinical significance of SVA in swine could not be determined (*7*,*8*).

In late July 2015, the Iowa State University Veterinary Diagnostic Laboratory (ISUVDL) received reports of 4 apparently unrelated cases of IVD affecting exhibition and commercial swine. The first 3 cases originated from unrelated farms located in southwest and central Iowa and were observed at 2 county fair exhibitions. The fourth case was observed in a commercial finisher farm in South Dakota. Affected animals exhibited acute lameness, anorexia, lethargy, and transient fever without associated mortality; they also exhibited coronary band hyperemia and vesicles, which occasionally progressed to cutaneous ulcers, as previously reported (*5*,*6*). Small vesicles were also evident on the snout, within the oral cavity, or both; these vesicles variably progressed to ulceration. No specific microscopic lesions beyond the ulcerative changes were present in specimens submitted to ISUVDL.

We collected vesicular lesion swab specimens and blood samples from all affected animals, and all tested negative for the viruses causing vesicular diseases mentioned previously (foot-and-mouth disease, swine vesicular disease, vesicular exanthema of swine, and vesicular stomatitis). No other common swine pathogens except SVA were detected at ISUVDL. By using a quantitative real-time reverse transcription PCR assay, we targeted a conserved region between the 5′ untranslated region and protein L (602–710 bp) and detected SVA RNA in vesicular fluids, epithelial scrapings of the snout, coronary band lesions, and/or hoof lesions with quantities ranging from 2 × 10^7^ to 1.2 × 10^11^ genomic copies/mL. We also identified the virus in serum and fecal samples, indicating SVA viremia and shedding. In a follow-up submission from the South Dakota premise, we detected SVA in nearly all of the tissues tested; inguinal lymph nodes and tonsils contained the highest SVA loads. Seroconversion to SVA in all affected swine was evident by indirect fluorescent antibody test titers ranging from 1:160 to 1:1,280 at 2–3 weeks after the clinical outbreak.

Our attempts to isolate the virus by using ST cells (ATCC CRL-1746; ATCC, Manassas, VA, USA) and NCI-H1299 (ATCC CRL-5803) (*8*) yielded cytopathic SVA isolates with titers up to 1 × 10^9^ PFU/mL from multiple vesicular lesion swabs or scrapings. We designated a representative isolate from each Iowa case as SVA15-39812IA, SVA15-40380IA, and SVA15-40381IA and the South Dakota case as SVA15-41901SD. Sequencing of viral protein (VP) 1 as previously described (*7*) demonstrated that each SVA isolate had a VP1 sequence identical to that of the virus in clinical specimens.

We obtained almost full-length genomic sequences (7,116–7,221 nt) of the 4 SVA isolates by using next-generation sequencing technology (*9*) and through de novo assembly (GenBank accession nos. KU051391–4). Sequence alignments showed that the isolates shared 98.9%–100% nucleotide identity with each other but diverged by 2.1%–2.2% from SVA isolate SVV-BRA-MG1-2015 (GenBank accession no. KR063107.1), by 3.9%–4.0% from SVA isolate 11-55910-3 (accession no. KC667560.1), and by 6.1%–6.4% from SVA isolate SVV-001 (accession no. DQ641257.1). Phylogenetically, the new US SVA isolates formed their own clade separated from all other SVA isolates (Figure, panel A). Such a branching out remained even when VP1 sequences, which are typically used for picornavirus phylogenetic analyses, were compared (Figure, panel B). All 4 isolates, along with VP1 sequences of SVA from 6 additional submissions from commercial farms in Iowa, Illinois, and South Dakota (2015044256SD, 2015046008IA, 2015046494IL, 2015047169IA, and 2015047271IL) and the Iowa State Fair (2015044662IA), were clustered (98.7%–100% identity) and separated from recent SVA isolates from Brazil (*6*) with 97.8%–98.0% identity. The viruses were further distant from other historical SVA isolates, showing 86.2%–95.7% identity.

**Figure:**
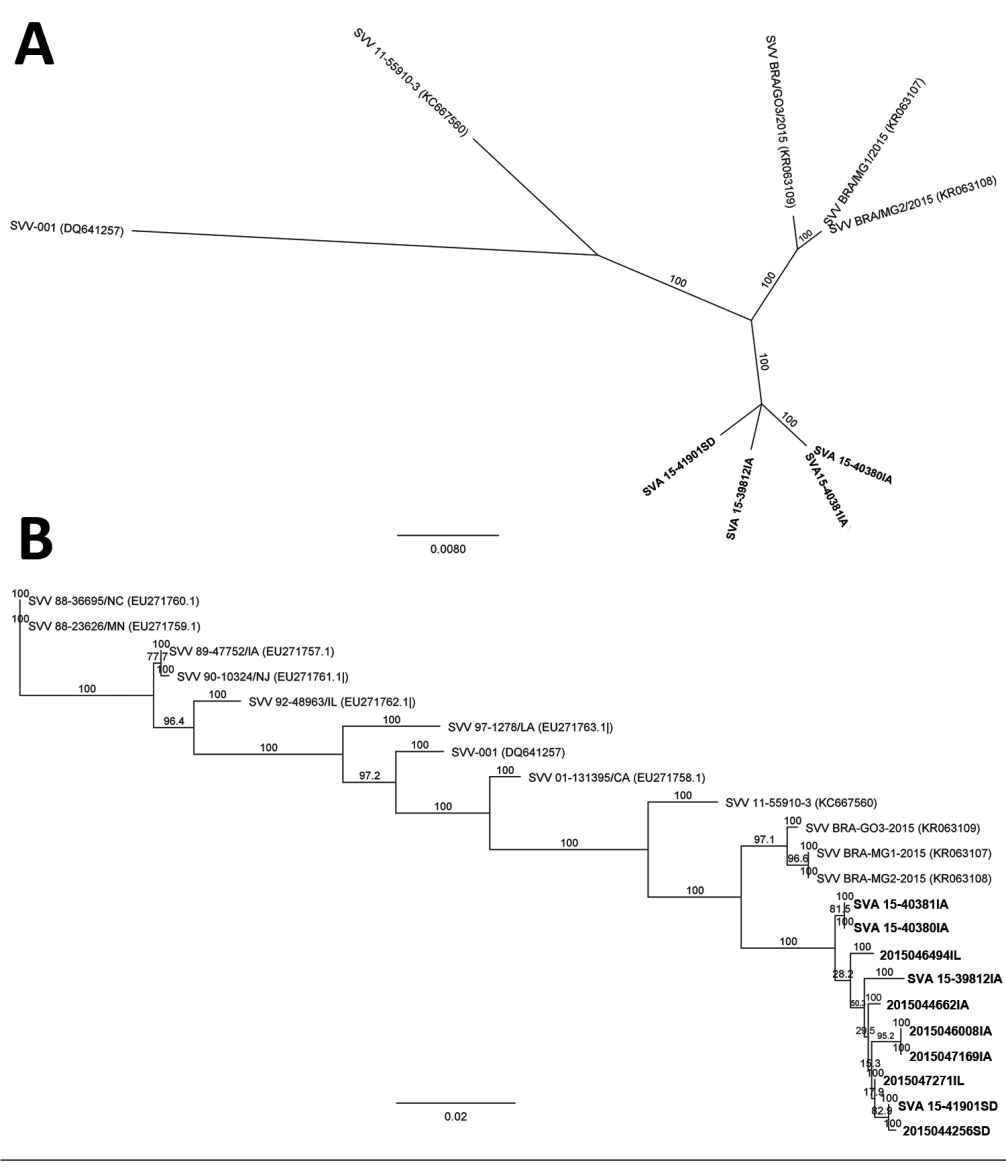
Phylogenetic relationships of 2015 US Senecavirus A (SVA) isolates (SVA15-39812IA, SVA15-40380IA, SVA15-40381IA, and SVA15-41901SD) with the prototype SVA isolate (SVV-001), a 2011 Canada swine SVA isolate (11-55910-3), and 2015 Brazil swine SVA isolates (SVV-BRA-G03-2015, SVV-BRA-MG1-2015, and SVV-BRA-MG2-2015). A) Full-length genomic sequences of 4 isolates from Iowa and South Dakota (bold) compared with reference isolates. B) Viral protein 1 sequences of 4 isolates from Iowa and South Dakota and 6 additional sequences from Iowa, Illinois, and South Dakota (2015044256SD, 2015044662IA, 2015046008IA, 2015046494IL, 2015047169IA, and 2015047271IL) (bold) compared with reference isolates. Trees were determined by using the neighbor-joining method with 1,000 bootstrap replicates. GenBank accession numbers for reference isolates are provided in parentheses. Scale bars indicate nucleotide substitutions per site.

Laboratory findings suggest that SVA infection was the etiology of these cases because no other common pathogen was detected across the cases examined; index swine were viremic, shed SVA in feces and nasal secretions, and seroconverted to the virus; a high level of SVA was present in areas with vesicular lesion; and evidence of disease spread among pen mates. SVA detected in these cases were genetically distinct from previously reported SVA, suggesting that the virus has evolved, possibly leading to higher adaption to swine and change in pathogenicity. Although SVA is not a new virus, numerous unrelated cases of vesicular disease at exhibitions and commercial farms within such a short period is unusual. The fact that swine producers in Brazil have experienced an epidemic of vesicular diseases, in which SVA similar to the recent US SVA was implicated, warrants further studies to characterize the pathogenesis and associated risk factors of this novel SVA in swine.
